# The Role of Complex Trauma and Attachment Patterns in Intimate Partner Violence

**DOI:** 10.3389/fpsyg.2021.769584

**Published:** 2022-01-25

**Authors:** Anna Maria Speranza, Benedetto Farina, Caterina Bossa, Alexandro Fortunato, Carola Maggiora Vergano, Luigia Palmiero, Maria Quintigliano, Marianna Liotti

**Affiliations:** ^1^Department of Dynamic and Clinical Psychology, and Health Studies, Sapienza University of Rome, Rome, Italy; ^2^Department of Human Sciences, European University of Rome, Rome, Italy; ^3^European Society for Trauma and Dissociation, Nijmegen, Netherlands

**Keywords:** intimate partner violence, attachment disorganization, complex trauma, childhood trauma, victimization, attachment trauma

## Abstract

**Objective:**

Even if the relationship between adverse childhood experiences and intimate partner violence (IPV) has already been established, there are no sufficient studies examining the relationships between these factors and attachment representations, specifically attachment disorganization. Thus, this study aimed to explore, in a sample of women who experienced IPV (a) the presence of interpersonal adversities during childhood, and (b) attachment representations, with a particular focus on disorganization.

**Methods:**

Women’s representations of attachment experiences were investigated through the Adult Attachment Interview, while the presence of various forms of interpersonal adversities during childhood was assessed using the Complex Trauma Questionnaire. The results of the IPV group (*n* = 98) were compared with those of women with no history of IPV (control group, *n* = 81).

**Results:**

Women in the IPV group showed higher values of multiple forms of adverse experiences within their caregiving system. They presented significantly higher rates of disorganized states of mind regarding attachment, indicative of a lack of resolution regarding traumatic experiences, and of disorganized working models resulting from complex trauma. Our results highlighted that, more than the presence of traumatic experiences, it is their irresolution – reflected in the disorganized states of mind regarding attachment at the Adult Attachment Interview – to be a significant predictor of IPV.

**Conclusion:**

These results suggest underline the significance of offering a trauma- and attachment-informed therapy to those who experience IPV. Such results could help both clinicians and researchers in formulating clearer guidelines for IPV interventions.

## Introduction

Intimate partner violence (IPV) encompasses abusive acts that cause pain or injury, such as physical or sexual violence, and an array of behaviors designed to control or intimidate, such as psychological aggressions and stalking. Those acts may be perpetrated by a current or former date, boyfriend/girlfriend, spouse, or cohabiting partner (Center for Disease Control and Prevention [CDC], 2021). Different types of violence generally coexist within the same relationship: most individuals with an history of IPV experience multiple forms of abuse over time (World Health Organization [WHO], 2012) with severe adverse effects on their mental and physical health (Dutton et al., 2006). Although violence may also be initiated by women, mutual within the couple, or found in same-sex relationships (Bates, 2016; Rollè et al., 2018), about 85% of all who experience IPV are women (Rennison and Welchans, 2000).

Despite its diffusion, the dynamics underlying IPV are still unclear. Indeed, IPV is a complex and stratified phenomenon that requires an articulated reading. Traditionally, it has been explained in terms of imbalanced dynamics of power and control between genders or of poverty, disempowerment, and social isolation (McCarthy et al., 2018). Recently, instead, a growing body of research (Doumas et al., 2008; Godbout et al., 2009; Dutton, 2011; Dutton and White, 2012; Levendosky et al., 2012; Craparo et al., 2014; Park, 2016; Pallini et al., 2017) has suggested that attachment theory is a useful framework to better comprehend IPV and how violence and intimacy can coexist within a relationship.

In this perspective, it is useful to introduce two concepts: complex trauma (Cook et al., 2005) and attachment disorganization (Bowlby, 1969; Solomon and George, 2011), since following West & George (West and George, 1999) we believe that it is helpful to consider attachment disorganization, more than insecure attachment, as a risk factor for IPV. Complex trauma refers to cumulative and interpersonal adverse experiences occurring at an early age, often within primary caregiving relationships. For this reason, it is often referred to using the expression “attachment trauma” (Isobel et al., 2017). Attachment trauma often leads to disorganized, contradictory and segregated internal working models (Bowlby, 1980; Blizard, 2003). Disorganized internal working models, in turn, result in the development of conflicting, chaotic and rapidly shifting representations of both self and others in which violence might be confused with love, possession with intimacy. For example, it has been argued that attachment trauma could lead to the *implicit* belief to deserve to be abused in intimate adult relationship, since such maltreatment somehow reflects one’s experiences within primary care systems, or since the abuser is perceived as someone that offer (even if in a perverted form) the attention and care lacked during childhood (Knox, 2016).

However, to our knowledge, there are not sufficient studies investigating the role of attachment disorganization and unresolved traumatic experiences in IPV, as highlighted in a recent meta-analysis (Spencer et al., 2021). Most studies on IPV and attachment have, in fact, focused on insecurity, often using self-report questionnaires that may not evaluate the role of attachment disorganization and early experiences adequately (Velotti et al., 2018).

Suggesting that attachment patterns could help us to better understand IPV does not mean that attachment is its primary cause (there are, of course, several intertwining factors at play, many of them also linked to cultural aspects and gender representations). However, referring to attachment theory seem useful to develop effective ways to inform IPV interventions, since it provides new ways to understand some of its underlying – and less investigated, perhaps because less recognized – dynamics, as it will be further discussed in this article. Such dynamics reside in deep, implicit processes which are not under the free and conscious control of the individual. Therefore, highlighting the importance of early attachment experiences should not lead to believe, in any way, that those who experience IPV are somehow responsible, and thus to blame, for what has happened to them – neither in their childhood nor in their adult relationships.

In this work, we investigated the presence of traumatic experiences and the quality of attachment representations in a sample of women with an history of IPV. We hypothesized that, compared to a control group, the IPV group would present a) more frequent and severe adverse childhood experiences and b) more disorganized states of mind regarding attachment.

## Aims

The present study aimed to investigate the role of significant risk factors for IPV related to early experiences with primary caregivers to identify some elements that, even if often overlooked, could represent an important focus during psychotherapeutic work with those who experience IPV.

We were interested in exploring in a sample of women with an history of IPV (a) the traumatic developmental context in which the co-occurrence of multiple types of adverse interpersonal experiences may have emerged, and (b) attachment representations regarding interactions within caregivers during childhood, with a particular focus on the presence of unresolved elements regarding traumatic experiences, and on indicators of a disorganized attachment during childhood.

## Materials and Methods

### Participants

Participants were 98 women with an history of IPV, recruited from support and shelter services in central and northern Italy. Inclusion criteria were a) being 18 years of age or older; b) having experienced violence by one or more intimate partners; c) having an appropriate knowledge of the Italian language. The age range was from 19 to 63 years old (*M* = 40.21, *SD* = 9.84). Exploring more thoroughly the characteristics of the abusive relationship through an *ad hoc* questionnaire, we found that 58% of women in the IPV group experienced multiple forms of maltreatment over time, and that the average length of the abusive relationship was 13 years (*SD* = 9.45). The relationship was still ongoing for 15% of women. In addition, 57% (*n* = 56) of women in the IPV group had children, born from the abusive relationship in 88% (*n* = 49) of cases. Moreover, 67% (*n* = 39) of children had witnessed episodes of domestic violence.

The control group was formed by 81 women recruited from obstetrics and gynecology clinics for checkup visits. Eligibility for participation in the control group included: (a) being 18 years of age or older, (b) absence of IPV, as evaluated through a clinical interview, and (c) an appropriate knowledge of the Italian language. The age range was from 25 to 62 years old (*M* = 33.09, *SD* = 7.64).

The socio-demographic characteristics of the two samples are shown in Table 1.

**TABLE 1:** Socio-demographic characteristics.

		IPV (*n* = 98)	Controls (*n* = 81)
Nationality	Italian	78% (*n* = 76)	97% (*n* = 79)
	Other	22% (*n* = 22)	3% (*n* = 2)
Level of education	Middle school	2% (*n* = 2)	3% (*n* = 2)
	High school	47% (*n* = 46)	27% (*n* = 22)
	University degree	48% (*n* = 47)	88% (*n* = 53)
	Post-graduate degree	3% (*n* = 3)	4% (*n* = 3)
Profession	Unemployed	13% (*n* = 13)	8% (*n* = 6)
	Temporary worker	19% (*n* = 19)	21% (*n* = 17)
	Housewife	1% (*n* = 1)	3% (*n* = 2)
	Employee	48% (*n* = 47)	43% (*n* = 35)
	Self-employed	19% (*n* = 19)	25% (*n* = 20)

All participants gave their informed consent for participation. All procedures performed in this study were in accordance with the ethical standards of the Italian Psychological Association and the 1964 Helsinki declaration and its later amendments. The protocol was approved by the Ethical Committee of the Sapienza University of Rome.

### Measures

#### Adult Attachment Interview (George et al., 1984)

The AAI is a semi-structured interview exploring adults’ attachment representations and designed to elicit memories of childhood experiences. AAI scoring system is based on the participant’s ability to produce coherent narratives, thus classifying the subject as Secure/Autonomous (F); Dismissing (Ds); Preoccupied (E); Unresolved with respect to loss or trauma (U); Cannot Classify (CC). Unlike Secure, Dismissing, and Preoccupied adults (organized classifications), individuals classified as Unresolved (U) show signs of disorientation and disorganization in the monitoring of reasoning or discourse during discussions of potentially traumatic events. Cannot Classify (CC) individuals show a global disruption of attachment strategies, oscillating between opposite and contradictory mental states (Ds and E). For the present study, U and CC transcripts were put together as “Disorganized” classifications. Considering the aims of this research, we decided to administer a version of the AAI containing an additional set of questions designed to explore more carefully a wide array of possible traumatic childhood experiences, following Madigan and colleagues (Madigan et al., 2012). Psychometric testing and meta-analyses have provided evidence of good stability and discriminant and predictive validity of the AAI in both clinical and non-clinical populations (van IJzendoorn, 1995; Bakermans-Kranenburg and van IJzendoorn, 2009). In the present study, each AAI transcript was rated by two certified coders blind to each other.

#### Complex Trauma Questionnaire (Maggiora Vergano et al., 2015)

The ComplexTQ is a 70-item scale for the retrospective assessment of interpersonal maltreatment during childhood (i.e., psychological, physical, and sexual abuse, physical and emotional neglect, rejection, role reversal, exposure to domestic violence, separations, and losses). The instrument is available in both a clinician- and a self-report version. In this study, we used the clinician-report one, applying it to AAI transcripts to evaluate the subject’s trauma history before age 15. The clinician evaluates both the presence and the frequency of such experiences. The latter is evaluated providing rates on a 4-point Likert scale (from 1 = never, to 4 = often, for each item except for the last one, which regards bereavements. Thus, the scoring – differentiated between the mother, the father, and another significant figure – allows detecting both the presence and the severity of each form of maltreatment. The ComplexTQ has shown good internal consistency of factors, convergent validity, and inter-rater reliability (Maggiora Vergano et al., 2015). The advantage in using this instrument consists in its ability to assess a broad range of childhood adverse experiences, including aspects that are often overlooked. In the present study, two certified coders (blind to each other) assessed the scores on the ComplexTQ scale.

## Statistical Analyses

To determine whether there was a significant difference between the frequencies of AAI categories within the two samples, we used the Chi-square distribution test and calculated both standardized and adjusted residuals. For the scores obtained on the ComplexTQ scales, *t*-tests were used to analyze the differences between groups after performing a Levene test. Due to the high number of *t*-tests performed, we calculated the false discovery rates (FDR) using the Benjamini–Hochberg (BH) method (Benjamini and Hochberg, 1995). Furthermore, a stepwise logistic regression was performed to explore which variables were the most significant in predicting IPV. The criterion for significance was set at *p* = 0.05. All statistical analyses were performed using the Statistical Package for Social Sciences (SPSS, version 27; IBM, Armonk, NY, United States).

## Results

Regarding the AAI classifications in the two groups, as shown in Table 2, the IPV sample was characterized by a remarkably high percentage (55%) of Unresolved (Ud) and/or Cannot Classify (CC) state of mind, indicative of some sort of disorganization in the women narratives. Such percentage was significantly higher than in the control group, while the presence of Free/Autonomous (F) states of mind – indicative of the ability to narrate attachment past experiences in a coherent and balanced manner – was significantly lower in the IPV group.

**TABLE 2:** AAI classifications in the IPV group and the control group.

	F	Ds	E	Ud/CC
Controls (*n* = 81)	52 (64%) (expected count = 36) *sr* = 2.6 z_*r*_ = 4.8	13 (16%) (expected count = 10) *sr* = 1.0 z_*r*_ = 1.4	6 (8%) (expected count = 6) *sr* = −0.0 z_*r*_ = −0.1.0	10 (12%) (expected count = 29) *sr* = 3.5 z_*r*_ = −5.9
IPV (*n* = 98)	28 (29%) (expected count = 44) *sr* = −2.4 z_*r*_ = −4.8	9 (9%) (expected count = 12) *sr* = −9 z_*r*_ = −1.4	7 (7%) (expected count = 7) *sr* = 0.0 z_*r*_ = −0.1.0	54 (55%) (expected count = 35) *sr* = 3.2 z_*r*_ = 5.9
χ^2^ = 36.973, *p* = < 0.001, df = 3, φ = 0.454				

*F, free/autonomous; Ds, dismissing; E, entangled/preoccupied; Ud, unresolved; CC, cannot classify.*

More specifically, women in the IPV group showed a significant over-representation of both insecure (Table 3) and disorganized states of mind (Table 4).

**TABLE 3:** AAI classifications (secure vs. insecure) in the IPV and control group.

	Secure (F)	Insecure (Ds, E, and Ud/CC)
Controls (*n* = 81)	52 (64%) (expected count = 36) sr = 2.6 z_*r*_ = 4.8	29 (36%) (expected count = 45) sr = −2.4 z_*r*_ = −4.8
IPV (*n* = 98)	28 (29%) (expected count = 44) sr = −2.4 z_*r*_ = −4.8	70 (71%) (expected count = 54) sr = 2.1 z_*r*_ = 4.8
χ^2^ = 22.771, *p* = < 0.001, df = 1, φ = 0.357		

*Ud, unresolved; CC, cannot classify.*

**TABLE 4:** AAI classifications (organized vs. disorganized) in the IPV and control group.

	Organized (F, Ds, and E)	Disorganized (Ud and CC)
Controls (*n* = 81)	71 (88%) (expected count = 52) sr = 2.6 z_*r*_ = 5.9	10 (12%) (expected count = 29) sr = −3.5 z_*r*_ = −5.9
IPV (*n* = 98)	44 (45%) (expected count = 63) sr = −2.4 z_*r*_ = −5.9	54 (55%) (expected count = 35) sr = 3.2 z_*r*_ = 5.9
χ^2^ = 35.293, *p* = < 0.001, df = 1, φ = 0.444		

*Ud, unresolved; CC, cannot classify.*

Concerning the presence of a disorganized state of mind, women in the IPV sample obtained heterogeneous classifications. Instead, in the control group, all subjects with a disorganized state of mind obtained only an Ud classification (often related to recent traumatic events, such as losses). As shown by the standardized residuals and adapted residuals values, the higher frequency of all disorganized classifications observed in the IPV group is statistically significant (see Table 5).

**TABLE 5:** Sub-types of disorganized states of minds at the AAI in the IPV and control group.

Disorganized state of minds				
	Organized	Ud	CC	Ud/CC
Controls (*n* = 81)	71 (88%) (expected count = 52)	10 (12%) (expected count = 18)	0 (0%) (expected count = 3)	0 (0%) (expected count = 8)
	sr = 2.6 z_*r*_ = 5.9	sr = −1.8 z_*r*_ = −2.8	sr = −1.6 z_*r*_ = −2.3	sr = −2.9 z_*r*_ = −4.2
IPV (*n* = 98)	44 (45%) (expected count = 63) *sr* = −2.4 z_*r*_ = −5.9	29 (30%) (expected count = 22) sr = 1.7 z_*r*_ = 2.8	6 (6%) (expected count = 3) sr = 1.5 z_*r*_ = 2.3	19 (19%) (expected count = 10) sr = 2.7 z_*r*_ = 4.2
χ^2^ = 39.336, *p* = < 0.001, df = 3, φ = 0.469				

*Ud, unresolved; CC, cannot classify.*

We also compared the classifications obtained by the IPV group to normative and clinical samples, both international (Bakermans-Kranenburg and van IJzendoorn, 2009) and Italian (Cassibba et al., 2013). The IPV group significantly differed from each sample (see Tables 6, 7).

**TABLE 6:** Comparison of AAI classifications obtained by the IPV group with those of normative and clinical samples.

	IPV	International normative sample*^a^*	International clinical sample*^a^*	Italian normative sample*^b^*	Italian clinical sample*^b^*
F	28 (29%)	392 (56%)	426 (21%)	508 (60%)	43 (17%)
Ds	9 (9%)	112 (16%)	389 (23%)	172 (21%)	79 (30%)
E	7 (7%)	63 (9%)	241 (13%)	92 (11%)	44 (17%)
Ud/CC	54 (55%)	126 (18%)	797 (43%)	70 (8%)	94 (36%)
		χ^2^ = 67.203 *p* = < 0.001	χ^2^ = 12.343 *p* = < 0.05	χ^2^ = 198.614 *p* = < 0.001	χ^2^ = 29.171 *p* = < 0.001

*^a^Bakermans-Kranenburg and van IJzendoorn, 2009.*

*^b^Cassibba et al., 2013.*

*F, free/autonomous; Ds, dismissing; E, entangled/preoccupied; Ud, unresolved; CC, Cannot classify.*

**TABLE 7:** Comparison of AAI classifications (organized vs. disorganized) obtained by the IPV group with those of normative and clinical samples.

	IPV	International normative sample*^a^*	International clinical sample*^a^*	Italian normative sample*^b^*	Italian clinical sample*^b^*
Organized	44 (45%)	567 (82%)	1056 (57%)	772 (92%)	166 (64%)
Disorganized	54 (55%)	126 (18%)	797 (43%)	70 (8%)	94 (36%)
		χ^2^ = 66.581 *p* = < 0.001	χ^2^ = 167.816 *p* = < 0.001	χ^2^ = 29.171 *p* = < 0.05	χ^2^ = 10.538 *p* = < 0.05

*^a^Bakermans-Kranenburg and van IJzendoorn, 2009.*

*^b^Cassibba et al., 2013.*

*F, free/autonomous; Ds, dismissing; E, entangled/preoccupied; Ud, unresolved; CC, cannot classify.*

Using the ComplexTQ, we were able to assess the presence and frequency of various forms of maltreatment during childhood. As shown in Table 8, women in the IPV group presented statistically significant higher values regarding almost any traumatic experience, except concerning paternal rejection, sexual abuse, separations from the mother, and losses.

**TABLE 8:** ComplexTQ scores in the IPV and control group.

		IPV (*n* = 98)	Controls (*n* = 81)	*t*	df	*p*	Benjamini-Hochberg adjusted *p-*value
Neglect	Mother	1.58 (0.65)	1.02 (0.11)	8.333	103.686	<0.001	**0.002**
	Father	1.53 (062)	1.16 (0.26)	5.335	135.017	<0.001	**0.009**
Reject	Mother	1.57 (0.70)	1.02 (0.16)	7.494	105.524	<0.001	**0.006**
	Father	1.66 (2.89)	1.10 (0.25)	1.736	173	0.084	0.84
Role reversal	Mother	1.33 (0.54)	1.01 (0.06)	5.78	100.203	<0.001	**0.004**
	Father	1.16 (0.38)	1.03 (0.08)	3.241	106.298	0.004	**0.006**
Psychological abuse	Mother	1.28 (1.05)	1.01 (0.08)	2.488	97.272	0.024	**0.033**
	Father	1.17 (0.37)	1.04 (0.11)	3.286	117.676	0.001	**0.002**
Physical abuse	Mother	1.27 (0.49)	1.02 (0.10)	5.009	104.835	<0.001	**0.002**
	Father	1.22 (0.49)	1.01 (0.08)	4.188	102.893	<0.001	**0.018**
Sexual abuse	Mother	1.06 (0.28)	1.01 (0.03)	1.700	98.15	0.092	0.118
	Father	1.06 (0.30)	1.10 (0.22)	–0.889	175	0.375	0.397
Domestic violence	Mother	1.56 (0.78)	1.02 (0.10)	6.779	99.453	<0.001	**0.003**
	Father	1.56 (0.78)	1.05 (0.22)	6.184	113.569	<0.001	**0.003**
Separations	Mother	1.08 (0.49)	1.01 (0.08)	1.323	97.333	0.189	0.227
	Father	1.28 (0.75)	1.01 (0.06)	3.429	94.367	<0.001	**0.005**
Losses	Mother	1.07 (0.46)	1.34 (0.95)	–2.269	111.963	0.25	0.281
	Father	1.11 (0.56)		–5.167	84	<0.001	**0.002**

*Bold values are the significant ones (p = < 0.05 or p = < 0.001).*

Following Finkelhor et al. (2007) and Murphy and colleagues (Murphy et al., 2014), we grouped both IPV and control subjects in two categories: poly-traumatized (≥4 forms of childhood traumatic experiences) and not poly-traumatized (<4 childhood traumatic experiences). The percentage of women with an history of poly-traumatization during childhood was considerably higher in the IPV group (51%) than in the control group (14%), suggesting the presence of complex trauma in women who later experienced some form of maltreatment within their adult romantic relationship (see Table 9).

**TABLE 9:** Presence of multiple forms of childhood trauma in the IPV and control group.

	≥4 forms of trauma	<4 forms of trauma
Controls (*n* = 81)	11 (14%) (expected count = 28) sr = −3.2 z_*r*_ = −5.3	70 (86%) (expected count = 53) sr = 2.3 z_*r*_ = 5.3
IPV (*n* = 98)	50 (51%) (expected count = 33) sr = 2.9 z_*r*_ = 5.3	48 (49%) (expected count = 65) sr = 2.1 z_*r*_ = −5.3
	χ^2^ = 27.67, *p* = < 0.001, df = 1, φ = −0.393	

Finally, a stepwise logistic regression was performed to explore which variables were the most significant in predicting the membership to IPV group (see Table 10). In the first step, we inserted as predictors those demographics variables that have been traditionally considered as potential risk factors for IPV: Nationality, Education and Profession. In the second step, we inserted the variable Multiple forms (>4) of childhood trauma. In the third and final step we inserted the variable Organized vs. Disorganized state of mind regarding attachment. Even if, in the second step, we observed that the variable Multiple forms (> 4) of childhood trauma was a significant predictor of IPV [*p* = 0.000; Exp(B) = 6.919], when the variable Organized vs. Disorganized state of mind regarding attachment entered in the model, this result was no longer significant. The final regression model obtained shows that both Nationality [*p* = 0.002; Exp(B) = 0.082] and Organized vs. Disorganized state of mind regarding attachment [*p* = 0.000; Exp(B) = 0.125] predict IPV: specifically, being Italian and having an organized state of mind regarding attachment significantly reduce the probability to belong to the IPV group. The model explains the 40% of variance and has the 73.3% of probability of correctly predicting the membership to IPV group (see Table 11).

**TABLE 10:** Stepwise logistic regression. Variables in the equation.

		*B*	Std. Error	Wald	df	*p*	Exp(B)	95% CI for EXP(B)	
								Upper	Lower
Block 0	Costant	0.389	0.163	5.697	1	0.017	1.476		
Block 1	Nationality	–2.246	0.759	8.760	1	**0.003**	0.106	0.024	0.468
(*R*^2^ = 0.120)	Costant	2.398	0.739	10.541	1	0.001	11.000		
Block 2	Nationality	–2.384	0.779	9.364	1	0.002	0.092	0.02	0.424
(*R*^2^ = 0.299)	Multiple forms (≥4) of childhood trauma	1.934	0.428	20.410	1	**0.000**	6.919	2.989	16.012
	Costant	1.919	0.754	6.474	1	0.011	6.812		
Block 3	Nationality (Italian)	–2.498	0.79	9.996	1	**0.002**	**0.082**	0.017	0.387
(*R*^2^ = 0.401)	Multiple forms (≥4) of childhood trauma	0.847	0.524	2.619	1	0.106	2.334	0.836	6.511
	Organized vs. Disorganized state of mind (Organized)	–2.076	0.559	13.778	1	**0.000**	**0.125**	0.042	0.375
	Costant	3.844	0.940	16.736	1	0.000	46.718		

*Block 1: Nationality–Education–Profession.*

*Block 2: Multiple forms (>4) of childhood trauma.*

*Block 3: Organized vs. Disorganized state of mind regarding attachment. Bold values are the significant ones (p = < 0.05 or p = < 0.001).*

**TABLE 11:** Classification Table (Block 3)*^a^*.

Observed			Predicted		
			IPV		Percentage correct
			No	Yes	
Step 1	IPV	No	48	15	76.2
		Yes	26	67	72.0
	Overall percentage				73.7

*^a^The cut value is 0,500.*

Compared with the control group, women in the IPV group presented significantly lower Free/Autonomous (F) classifications at the AAI and significantly higher rates of Unresolved (Ud) and/or Cannot Classify (CC) classifications (i.e., of disorganized states of mind). Their interviews were strongly characterized by unprocessed traumatic experiences within their history. Besides, they showed heterogeneous disorganized classifications. While the control group presented only Unresolved classification regarding losses or traumas (often occurred in recent years), a significant part of disorganized subjects in the IPV group received a CC or both an Ud and CC classification. The CC classification reflects a kind of disorganization considerably more pervasive than that found in Ud subjects, since it involves a *global* breakdown in the organization and/or maintenance of a discourse strategy when discussing attachment experiences (Hesse, 1996).

More than the abnormal absorption and lapses of Ud subjects, the incoherence and conflicting strategies of CC subjects are likely the consequence of the activation of disorganized internal working models [IWMs; (Bowlby, 1969, 1980)] resulting from complex trauma (Liotti, 2004; Speranza et al., 2017). In the presence of disorganized IWMs, individuals may develop conflicting and chaotic representations of themselves and their relationships, confusing violence and love, abuse and intimacy (Meares, 2013).

The results of the ComplexTQ highlight how women in the IPV group frequently presented adverse interpersonal experiences during childhood, regarding not only physical or psychological abuse but also more “subtle” forms of trauma such as role reversal, neglect, and rejection. Moreover, such experiences seemed to be of multiple forms, indicating the presence of complex trauma. It seems noteworthy that the IPV group did not differ from controls concerning paternal rejection, while almost any other kind of traumatic experience regarding the father was significantly more frequent among IPV women (with the notable exception of sexual abuse). The presence of a father who was slightly less rejecting than the other attachment figure, and who may have brought the child to assume an inappropriate parental or spousal role (as inferred through the scores at the paternal role reversal scale), could have led to a mental representation regarding relationships with male figures characterized by the belief that women must be excessively caring and acquiesced to earn attention and care. Indeed, disorganization and experiences of role reversal in childhood can lead individuals to be unable to assert their needs in interpersonal relationships, even when abusive (Hennighausen and Lyons-Ruth, 2005).

Taken together, the ComplexTQ and AAI results show that women in the IPV group not only presented more frequent and multiple forms of childhood traumatic experiences, but also that such experiences had not been adequately resolved and integrated. As shown by the logistic regression (Table 9), more than the mere presence of traumatic experiences, is their irresolution – reflected in the disorganized states of mind regarding attachment at the AAI – that represents a significant predictor of IPV. Indeed, the presence of unresolved traumatic experiences compromises the ability to integrate sensory, emotional, and cognitive processes (Cook et al., 2005), leading to mentalization deficits (Luyten et al., 2020) and to a fragmented sense of self (Schore, 2003; Fisher, 2017). It also impairs self-esteem and the sense of agency and mastery (Barnum and Perrone-McGovern, 2017), and may create an overall annulment of one’s desires and needs. It may lead to difficulties in social communication, affect regulation, and coping with stressors (Porges and Dana, 2018; Kuzminskaite et al., 2020), therefore altering emotional tolerance and appraisal, particularly regarding danger and threat (Herringa, 2017). Such alterations have already been found in women who experienced IPV (Clauss and Clements, 2021). The annulment of one’s subjectivity, the diminished awareness of one’s own and others’ emotions observed in those who experience IPV – and that we can consider as risk and maintenance factors – could represent defensive strategies developed by those women already during childhood to protect themselves from the pain and incomprehensibility of their experiences (Foa and Hearst-Ikeda, 1996), and still active in adulthood due to a lack of recognition and integration in their history, as shown by AAI classifications. This factors thus need to be specifically addressed during psychotherapeutic work.

The presence of disorganized state of minds regarding attachment in the IPV sample suggests that these women had attachment figures incapable of responding adequately to their distress and who probably became frightened and/or frightening in response to their requests for care. During childhood, these women may have perceived that the expression of their needs could provoke rejection, abandonment, or punishment. The presence of multiple forms of maltreatment within their primary care experiences could have led these women to a representation of themselves as worthless and unlovable, deserving of their maltreatment, since their childhood. Acknowledging the intentions of the abusive parent, someone from whom the child depends upon, could be indeed further traumatizing (Knox, 2016). Thus, the abuse and maltreatment experienced during childhood could have been denied in the attempt to maintain psychological integrity, resulting in a similar tendency to not be able to fully recognize the abuse experienced later in adult intimate relationship, producing a continuous cycle of trauma and violence (Alexander, 2015).

Moreover, the presence of disorganized IWMs often leads to a paradoxical representation of the attachment figure as the source of both the comfort and love the child needs to ease their pain (i.e., as a rescuer) and of an ongoing fear (i.e., as a persecutor), while the self is represented as helpless [i.e., as a victim (Main and Hesse, 1990; Liotti, 2004, 2006)]. It has been proposed (Liotti, 2004) that in disorganized individuals, these three roles – persecutor, rescuer, and victim – are continuously and rapidly shifting, leading to painful, contradictory, and incompatible representations of self and others, as well of what relationships are. Disorganized children also construct a representation of themselves as somehow evil, responsible of the caregiver’s fear (i.e., self as persecutor) and of their caregiver as helpless, fragile (i.e., other as a victim). Finally, there is another simultaneous and conflicting representation of self-with-other in disorganized children, one in which the self is seen as able to comfort the caregiver (i.e., as a rescuer), while the latter remains weak or powerless (Liotti, 2004). Such conflicting and rapidly shifting representations may have continued to be activated within other intimate relationships, creating a risk and maintenance factor for IPV.

In summary, the absence of attachment figures able to respond to their needs and comfort them in times of distress could have brought women in the IPV group to have an implicit and automatic mistrust toward intimate others. These women could also have developed the belief that all relationships will replicate what experienced during childhood – with the self seen as void, deprived of value, and the other as hostile/frightening or helpless/frightened and yet both as in need of receiving care and attention and as the only source of comfort and security.

The fact that the AAI classifications obtained by the IPV group were significantly different from both normative and clinical sample (and characterized by a remarkably high percentage of disorganized state of minds) suggest that this population possesses unique characteristics regarding attachment experiences and representations. While there is a large body of research investigating the role of attachment insecurity in IPV, the scarcity of studies thoroughly examining attachment disorganization (Spencer et al., 2021) appears therefore surprising. The results of our work confirm those obtained by the few other preliminary studies that have explored attachment representations in women with an history of IPV using the AAI or other projective measures (Alexander, 2009; Pallini et al., 2017; Condino et al., 2020).

We would like to conclude our discussion emphasizing that disorganized attachment is not the only risk factor for IPV. As already mentioned in the introduction, IPV is a complex and multi-layered phenomenon in which several factors are at play, and none of them should be overlooked. Moreover, certainly not everyone who has a disorganized state of mind will experience violence in adult intimate relationship, nor everyone who experience it has unresolved attachment trauma – as shown by the results of this study.

However, our results shed light to some important potential risk factors for IPV, as well as on what could be protective factors for this phenomenon. Indeed, they show that more than the presence of childhood trauma is the fact that such experiences have remained unresolved and not integrated (as indicated by the presence of a disorganized state of mind) to be predictive of IPV. On the contrary, the presence of an organized state of mind significantly reduces the probability to belong to the IPV group. We would like to remark that organized states of mind at the AAI do not reflect an absence of adverse childhood experience; rather, they are indicative of the interviewee’s ability to organize their experience (regardless of its quality) in a coherent manner. These considerations suggest some clinical implications.

## Clinical Implications

A recent review (Trabold et al., 2020) has emphasized how IPV interventions are still characterized by much heterogeneity regarding theoretical backgrounds, perspectives, and techniques. This makes it difficult to compare them and individuate factors that can improve the physical and mental health of those who experience IPV (Condino et al., 2016). Moreover, even if lately trauma-focused individual interventions seem to be increasing, they still mainly focus on the recent traumatic experiences IPV victims suffered.

Our findings highlight the importance of offering to those who experience IPV a trauma- and attachment-informed therapy, helping them to recognize their early adverse experiences and how they could have played a role in their abusive experiences within adult romantic relationships. In this way, we can foster their ability to reflect on themselves’ and others’ behaviors and mental states, as well as their ability to integrate previous experiences in a coherent manner: all factors that may, *per se*, represent a protective factor regarding the vulnerability to experience IPV.

The presence of unresolved and not integrated adverse attachment experiences could have led women who later experienced IPV to feel an implicit and yet pervasive mistrust toward anyone who offers them care and attention (Knox, 2016), as well as to deficits in reflective functioning and affective mentalization (Jurist, 2018; Luyten et al., 2020). If not taken into consideration, these factors may create impasses during psychotherapy or other supportive interventions offered to those who experience IPV, thus making them ineffective. To prevent ineffective treatments could thus be necessary to focus on relational variables of psychotherapy, such as the therapeutic alliance, and using parallel integrated interventions, as a way of both coping with the consequences of disorganized attachment followed by relational trauma and dealing with self disturbances (Liotti and Farina, 2016; Monticelli and Liotti, 2021).

## Limitations and Final Remarks

One limitation of this study was its relatively small sample size. Besides, it could be helpful to consider other aspects of psychological functioning related to attachment patterns, such as mentalization and epistemic trust. It would also be interesting to examine if the abuses perpetrated by caregivers are processed and remembered differently from the ones committed by other figures.

Furthermore, it would be important to investigate more thoroughly how different factors and variables interplay in creating a vulnerability for IPV.

Finally, we want to remark that even if talking about the dynamics underlying IPV can be perceived as a form of victim-blaming, our aim is quite the opposite. Recognizing that there are factors that make some individuals more vulnerable to victimization is not equivalent, in any way, to condemn them for being mistreated, since the core aspect of such factors is that they are not under conscious and free control but reside in deep implicit processes. By recognizing these factors, we can help IPV victims break the vicious cycle of intergenerational transmission of trauma.

## Data Availability Statement

The data that support the findings of this study are available on request from the corresponding author.

## Ethics Statement

The studies involving human participants were reviewed and approved by Ethical Committee of Sapienza University of Rome. The patients/participants provided their written informed consent to participate in this study.

## Author Contributions

AS and BF: conceptualization of the study, supervision, project administration, and writing (review and editing). CB, CM, and LP: investigation. AF: analysis of data and writing (review and editing). MQ: analysis of data and methodology. ML: investigation, analysis of data, and writing (original draft). All authors contributed to the article and approved the submitted version.

## Conflict of Interest

The authors declare that the research was conducted in the absence of any commercial or financial relationships that could be construed as a potential conflict of interest.

## Publisher’s Note

All claims expressed in this article are solely those of the authors and do not necessarily represent those of their affiliated organizations, or those of the publisher, the editors and the reviewers. Any product that may be evaluated in this article, or claim that may be made by its manufacturer, is not guaranteed or endorsed by the publisher.
